# Anatomical study of the inferior extensor retinaculum and the oblique superolateral band: implications for the Brostrom-Gould procedure

**DOI:** 10.1186/s12891-021-04932-z

**Published:** 2022-01-04

**Authors:** Guanghui Zeng, Qi Liu, Dongming Cui, Chao Liang, Chunsheng Tao, Jinzhu Zhao

**Affiliations:** 1 Qingdao University, Qingdao, Shandong China; 2 NO.1 District Department of Orthopedics, No. 971 Army Navy hospital of People’s Liberation, Qingdao, Shandong China

**Keywords:** Inferior extensor retinaculum, Anatomy, Ankle, Brostrom-Gould

## Abstract

**Purpose:**

The Brostrom-Gould procedure is currently the gold standard surgical choice for the treatment of chronic ankle instability; it can significantly improve ankle function and stability in patients. However, recent studies have reported doubts regarding the feasibility of the inferior extensor retinaculum (IER) after Brostrom-Gould and therapeutic effects compared with the Brostrom procedure. The purpose of the present study was to observe the anatomical characteristics of the lateral part of the IER using cadaveric bodies in order to guide the surgical operation of chronic ankle instability.

**Methods:**

Twenty-three cadaveric ankles were dissected. The morphology of the IER and its internal structure was observed and recorded for each ankle. The shortest distance between the Stem ligament of the IER and the anterior fibular periosteum (AFP) was measured and recorded, then attempts were made to suture the Stem to the AFP.

**Results:**

Twelve of the cadaveric ankles were observed as having an oblique superolateral band (OSLB) that had a tough texture upward of the lateral IER connecting with SL, as are the characteristics of the oblique superolateral band (OSLB) reported in previous studies. The inner and outer membrane of the OSLB were connected with inner and outer membrane of Stem. The average value of the distance between the Stem and AFP was 11.60 ± 2.71 mm, and the maximum and the minimum distance were 19.04 mm and 6.53 mm, respectively. The P -value (*P* = 0.2) resulting from a single sample K-S test confirmed that the distribution of distances conformed to normality. None of the SL in the study could be sutured to the AFP.

**Conclusion:**

The OSLB of the IER has a tough texture and connects with the Stem, and has the potential be utilised in the Brostrom-Gould procedure. However, we do not recommend utilization of the Stem in this operation regardless of the distance between the AFP and the Stem. When the Stem cannot be used to enhance repair in this operation, other solutions can be used for strengthening and to protect the repaired ATFL.

## Background

Ankle sprain has a high prevalence in the general population, over 70% of people have been reported to have had a sprain in their lifetime [1, 2]. Some non-surgical methods can reduce the incidence of chronic ankle instability and although the majority of patients have accepted standard conservative treatment, approximately 20% of them still suffered chronic lateral chronic ankle instability [3, 4]. Recommendations of surgical treatment thus were proposed by many orthopaedic surgeons for whom conservative treatment failed. Brostrom [5] reported for the first time a surgical method that involved suturing the cracked anterior talofibular ligament (ATFL), Gould [6] then modified this process utilizing the inferior extensor retinaculum (IER) to fix the anterior fibular periosteum with the aim of strengthening the repaired ankle.

Brostrom-Gould is currently the gold standard surgical choice for the treatment of chronic ankle instability; it can significantly improve ankle function and stability in patients and clinical studies have found that the Gould process achieved excellent results [7–9]. Augmented repair with IER provides initial protection for the repaired ATFL and improves joint rotation function [10]. However, one cadaveric biomechanical study claimed that there were no significant differences between ankles operated on using the Brostrom versus the Brostrom-Gould procedure [11]. Recently published anatomic studies found that only IER with an oblique superolateral band could be repaired using Brostrom-Gould, whilst IER with only Stem or frondiform ligament could not [12, 13].

The purpose of present study was to observe the anatomical characteristics of the lateral part of the inferior extensor retinaculum of cadaveric ankles to guide the surgical operation of chronic ankle instability.

## Materials and methods

**Fig. 1:**
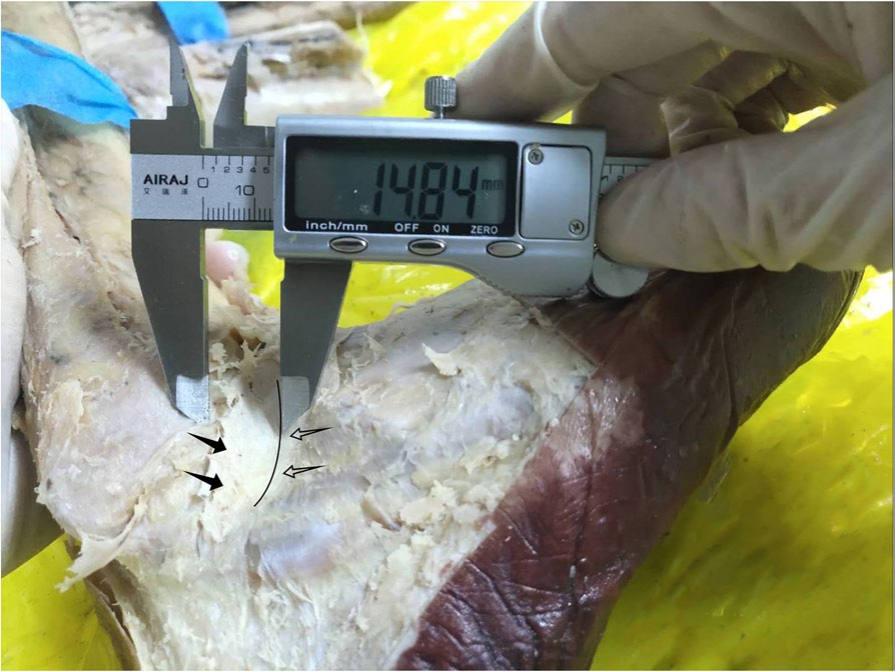
Measurement of distance between the stem ligament (SL) of the IER and the anterior fibular periosteum (AFP). OSLB (black arrow); stem ligament (hollow arrow); the boundary between Stem and OSLB (black line)

Twenty-three ankle specimens frozen and fixed in formalin were used for this study. The specimens were provided by the Anatomy Department of Qingdao University. All dissections were performed by the same researcher. After the specimens were thawed at room temperature. An appropriately sized skin dissection window was created at the anterolateral region of the ankle before skin, blood vessels and nerves were removed. Stem of IER was easily identified because of the white fiber bundle and thickness. The morphology of the IER of the lateral ankle was observed and the presence or absence of an oblique superolateral band (OSLB) was recorded. Three different researchers measured the closest distance between the anterior peroneal periosteum and the stem ligament of the IER using a vernier caliper (Fig. 1) at natural 90^o^ angle. Next, attempts were made to suture the Stem to the anterior fibular periosteum (AFP) using a non-absorbable suture (Holycon NO.0) at this position. Average, maximum, minimum distances was calculated. The conditions for failure were the Stem being torn or the suture being broken. All measurements were processed by three independent observers.

### Statistical analysis

For a statistical analysis, the single sample Kolmogorov-Smirnov Test was used to evaluate normality. Average, maximum and minimum distances were calculated. The intraclass correlation efficient (ICC) among three observers was calculated. A *P* value of > 0.05 was considered to represent normal distribution. All statistical analyses were performed by a statistician using version 26.0 IBM SPSS statistical software.

## Results

### Morphology of lateral IER

**Fig. 2:**
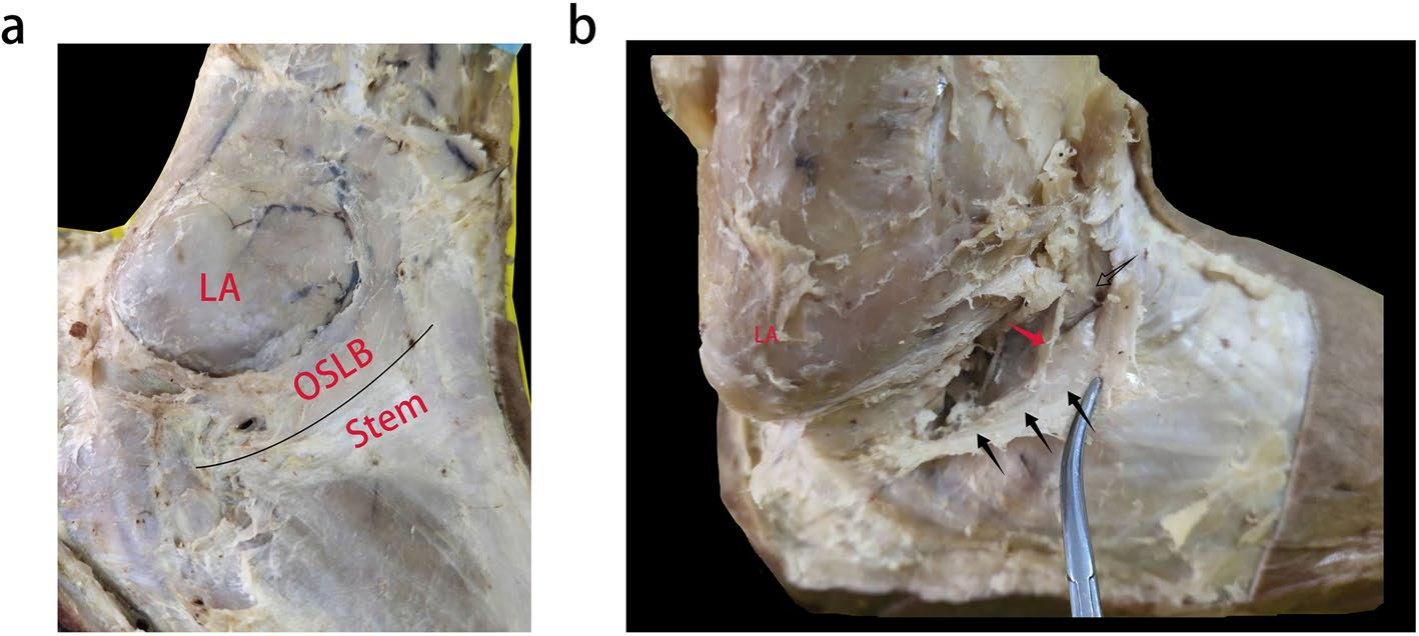
The relationship between the OSLB and surrounding tissue from an inner and outer aspect. The’ OSLB is connected with the upper part of the Stem (**a**). The inner membrane of the OSLB is connected to inner membrane of the Stem, the intermedial root of the IER is attached to the inner membrane (**b**). Lateral ankle (LA.), stem ligament (Stem), Inferior extensor retinaculum (IER), The boundary between the Stem and OSLB of IER (black line), Intermedial root of IER (red arrow), OSLB (black arrow), Extensor digitorum tendon (hollow arrow)

**Fig. 3:**
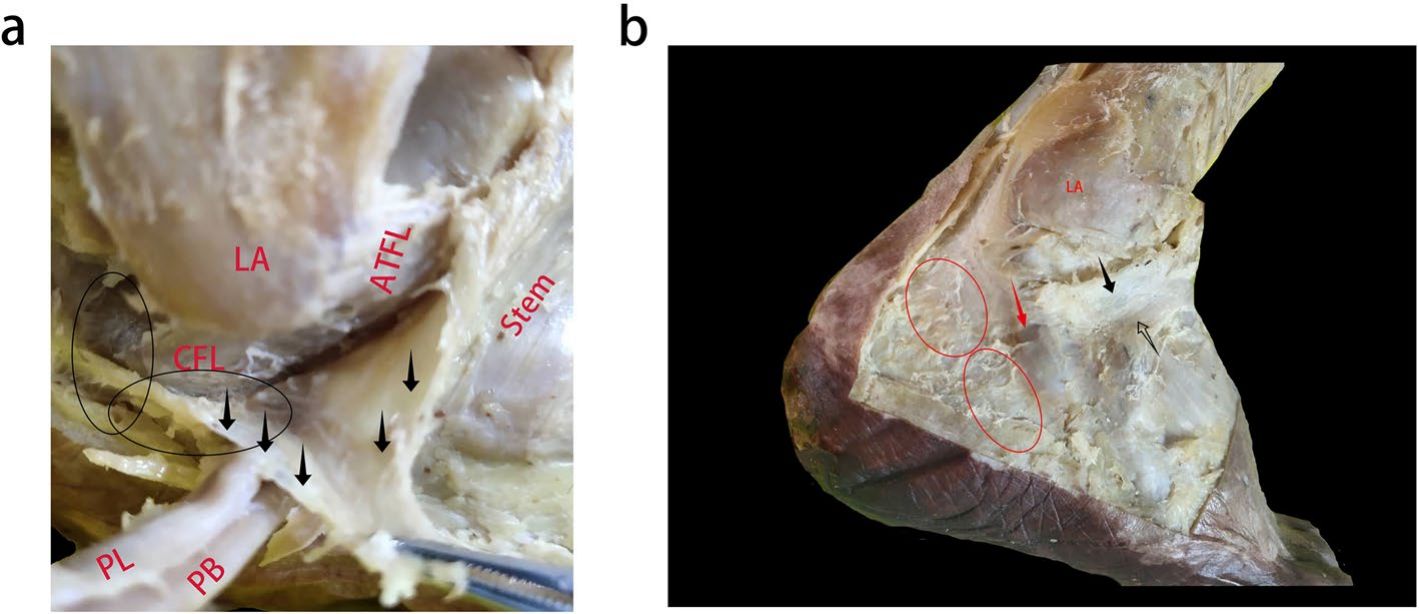
The footprint of the inner and outer OSLB on the lateral calcaneus. The inner footprint located around the CFL (**a**). The outer membrane of the OSLB was connected to outer membrane of Stem, crossing the sheath of peroneal tendon and ending at the lateral wall of the calcaneus surface (**b**). Lateral ankle (LA), Calcaneofibular ligament (CFL), Anterior talofibular ligament (ATFL), Peroneal longus tendon (PL), Peroneal brevis tendon (PB), OSLB (black arrow), Extensor digitorum tendon (hollow arrow), The footprint of outer membrane (red circle), stem ligament (hollow arrow), The peroneal tendons (red arrow)

**Fig. 4:**
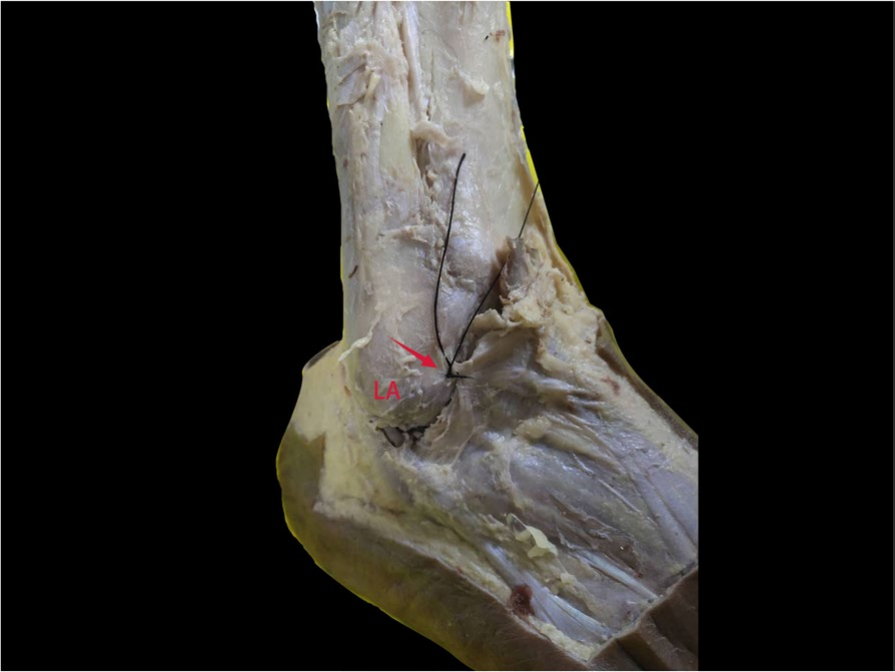
Aspect of the OSLB. TT could be easily sutured to the anterior fibular periosteum (red arrow). Lateral ankle (LA)

**Fig. 5:**
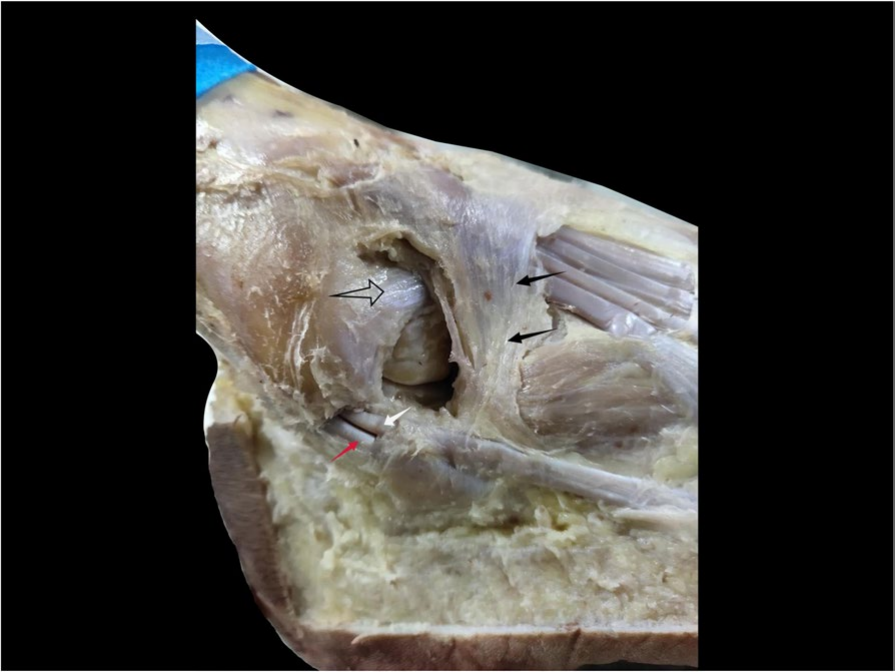
Lateral ankle with IER. Only one stem ligament could be identified (black arrow). Anterior talofibular ligament (hollow arrow), Peroneal longus tendon (red arrow), Peroneal brevis tendon (white arrow)

Among the 23 specimens dissected in this study, 12 cadaveric ankles had a tough texture to the structure upward of the lateral IER and its connection to the stem ligament (Fig. 2a). These tissues were consistent with having an oblique superolateral band (OSLB). Previously, Dalmau [12] categorised this type of IER as X-shaped. The inner membrane of the OSLB was connected to the inner membrane of the Stem, whilst the intermedial root of the IER was attached to the inner membrane of the OSLB, which formed a loop with the membrane around the extensor digitorum tendon (Fig. 2b). The inner membrane of the OSLB ended at the lateral wall of the calcaneus, close to the calcaneofibular ligament (CFL) (Fig. 3a). The outer membrane of the OSLB was connected to the outer membrane of the Stem, crossing the sheath of peroneal tendon and ending at the lateral wall of the calcaneus surface (Fig. 3b). In these cases, OSLB could be easily sutured to the anterior fibular periosteum (AFP) (Fig. 4). In the rest of the specimens (11 ankles), the Stem was identified with white fibers and no OSLB was observed (Fig. 5).

### Distance between stem ligament and anterior fibular periosteum

**Fig. 6:**
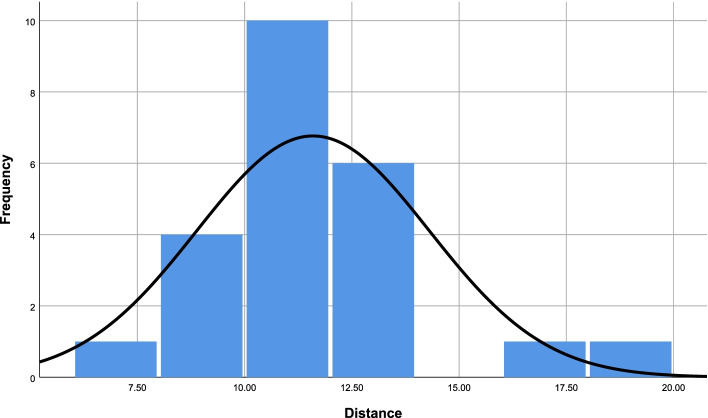
The frequency distribution histogram of the measured distances

The average distance between the Stem and the anterior fibular periosteum (AFP) was 11.60 mm, and the maximum and minimum distances were 19.04 mm and 6.53 mm (Table 1), respectively. *P* > 0.05 (*P* = 0.2) in a single sample Kolmogorov-Smirnov test suggested that the distribution of the distances conformed to normality (Fig. 6). There were no instances in which the stem ligament could be sutured to the anterior fibular periosteum. (Since all stem ligaments were torn by the suture).

**Table 1:** The distances between the Stem and the AFP as measured by three independent observers (mm)

Specimen	Observer1	Observer2	Observer3	Average
1	13.51	13.03	13.02	13.19
2	13.16	12.80	14.68	13.55
3	16.82	16.74	16.73	16.76
4	10.90	10.37	9.70	10.32
5	8.61	9.61	9.69	9.30
6	10.82	10.30	11.00	10.71
7	6.28	7.04	6.26	6.53
8	11.35	11.55	11.19	11.36
9	10.59	9.95	10.69	10.41
10	11.98	11.79	10.75	11.51
11	11.84	11.31	11.59	11.58
12	12.22	11.91	11.69	11.94
13	8.27	8.51	8.12	8.30
14	11.79	11.42	10.90	11.37
15	9.54	9.59	9.12	9.42
16	13.31	12.68	11.72	12.57
17	8.75	8.13	8.71	8.53
18	10.65	10.11	9.64	10.13
19	18.84	19.14	19.13	19.04
20	14.51	12.31	13.01	13.28
21	14.63	13.92	13.16	13.90
22	12.71	12.43	12.53	12.56
23	10.47	10.57	10.33	10.46
Maximum	–	–	–	19.04
Minimum	–	–	–	6.53
Average	–	–	–	11.60 ± 2.71
ICC		0.968		*P* < 0.001

## Discussion

Variation in IER characteristics in the different ankle samples was exclusively identified in the lateral region, specifically in the white fiber bundle. Previous studies considered IER as Y-shaped with only a stem ligament (or frondiform ligament) at the lateral region [14, 15]. Three roots of IER were consistently described in previous studies: lateral, intermedial and medial [16–18]. The medial root of an IER can be divided into a medial and lateral component and the medial component is blended with fibers of the interosseous talocalcaneal ligament (ITCL). Where the ITCL inserts into the calcaneus a V-shape is formed. Meanwhile, the lateral component ran almost vertically towards the calcaneus, and is inserted into the lateral border of the tarsal sinus [19]. The intermediate root of the IER is widely attached to the calcaneus [20], whilst the lateral root blended with the peroneal tendon sheath [15]. Wood Jones and Kuhlmann et al. described that only the sling fibers of the retinaculum act as pulleys for the frondiform ligament, and the groove beneath an IER formed a loop to prevent the extensor digitorum tendon from dislocating [21].

Recently, reports of X-shaped IER have been rising, ABU-HIJLEH [21] identified an X-shaped cruciate structure in the deep fascia of IER in 9 among 14 specimens, consistent with a Weitbrecht ligament which Jones reported [22]. Dalmau [12] also identified that 17 of the 21 specimens presented with an X-shaped IER and only 4 presented a Y-shaped IER, it was however considered that the oblique superolateral band of X-shaped IER was too weak for Brostrom-Gould to be used. We observed that in 23 ankle specimens, 12 cases were identified as having an oblique superolateral band (OSLB) upward of the lateral IER region, with a connection to the stem ligament. In these cases, the OSLB had a tough texture and clear boundary between the stem ligament which was observed as white fibers (Fig. 2a). These findings were similar to the oblique superolateral band described by Dalmau [12, 13], in which it was stated that previous studies proceeding Brostrom-Gould might make use of the sural fascia to augment.

Previous studies did not report information regarding the inside of the OSLB within the sinus tarsal, or only studied the relationship between the Stem and extensor tendon. In cases in this study, the inner membrane of the OSLB was connected to the inner membrane of Stem, and the intermedial root of the IER was attached to the inner region of the OSLB structure, forming a loop with the membrane around the extensor digitorum tendon (Fig. 2b). The inner membrane of the OSLB ended at the lateral wall of the calcaneus, close to calcaneofibular ligament (CFL) (Fig. 3a). Meanwhile, the outer membrane of the OSLB was connected to the outer membrane of the Stem, crossing the sheath of the peroneal tendon and ending at the lateral wall of the calcaneus surface (Fig. 3b). Both the inner and outer membrane were connected to the bone with good strength. Therefore, to strengthen the repair of the ligament, some orthopaedic surgeons created an IER flap as a graft, by using part of the IER tissue and fixing it to the fibular canal, the follow-up effect after surgery was considerable [23, 24]. In a recent study, Pintore et al. [25] used an IER flap combined with fibular periosteal flap to treat patients with failed chronic ankle surgery. Patients had a 92.3% satisfaction rate at the last follow-up.

An IER of the ankle can be described in terms of 3 functional layers in histology. A dense collection of fibroblasts and collagen fibers dominate the middle layer, oriented primarily perpendicular to the direction of the underlying tendons [26]. An OSLB with minor toughness was identified very close to the anterior fibular periosteum and even blended with it; this OSLB could be easily sutured to the fibular periosteum (Fig. 4). However, since biomechanical analysis was not carried out, it was impossible to determine its strength or whether it met the conditions of augmentation. On the contrary, the Stem is a multi-layered structure that is inserted in the lateral calcaneus and is blended with the peroneal tendon sheath, grooves were formed in upside of the extensor tendon of the foot to allow the extensor tendon to slide freely and prevent dislocation. The white fiber bundles of the Stem can be seen by the naked eye, it is therefore ideal tissue for a ligament augmentation operation [15, 21].

Therefore, we further measured the shortest distance between the Stem and the anterior periosteum of the fibula to evaluate the maximum distance for achieving a successful Gould operation. The average value of the distance between the Stem and the anterior fibular periosteum was 11.60 mm, whilst the maximum and the minimum were 19.04 mm and 6.53 mm, respectively (Table 1). The distribution of the measured distances conformed to normality. Furthermore, we used silk sutures to suture the Stem of the IER to the periosteum as much as possible, and found that in all cases the Stems could not be sutured to the fibula (since all Stems were torn by the suturing process). This finding suggests there was too much tension on the Stem. Jeong [8] measured the shortest distance between the IER and the distal anterior fibular periosteum (DAFP) during repair surgery for patients with chronic ankle instability. The average distance was 9.8 mm in their study. Jeong et al. believed that a distance of more than 18 mm suggested the DAFP could not be used for the augmentation process. If the distance was longer than 18 mm, excessive dorsiflexion was required that may cause the IER to tear immediately after surgery. The average of distance measured in our study was similar to in this previous study but in ours none of the IER could be sutured to the fibular periosteum. The present study revealed that the distance is perhaps not the most important factor determining the success of the procedure, regardless of how far the Stem was from the periosteum. In present study, we do not recommend use the Stem to strengthen ATFL repair.

We analyzed the reasons for inability of the Stem sutured to the fibular. White fibers were identified in Stem of the IER, these fibers may be one of causes of the poor deformability of the IER, and, therefore, we suggest that the stem ligament cannot be sutured to the anterior fibular periosteum. Another reason might be that specimens had been stored in formalin for many months, causing the ankles in our study to be quite different from those in living bodies since the formalin further led to stiffness and deformability decline so that IER could be easily torn. Finally, we consider that that the suture could have been too fine.

Due to anatomical factors, in some patients, the IER cannot be used for augmentation for repair of a damaged ATFL, this may subsequently suggest that there was no significant difference in biomechanics between the Brostrom-Gould and Brostrom procedures [11]. When the IER cannot be used for enhancement surgery, in addition to the aforementioned IER flap for ATFL repair and enhancement surgery [23, 24], some orthopaedic surgeons have also used the fibular periosteal flap for repair and enhancement, and have achieved good results [27]. Cho et al. [28] used a suture tape that was also utilized to strengthen the repair of the ligaments; this had significant positive clinical effects.

There are some limitations to present study. First of all, due to the small sample size in our study, some statistical errors may be unavoidable. Furthermore, the proportion of the two types of IER in our study differed from previous studies, which may have affected the results. Secondly, judgement regarding whether the fascia was removed entirely depended on the experience, sense or even vision of each observing individual, therefore some subjective interference possibly impacted the identification of OSLB. Thirdly, the Stem displays variety in its angle to the horizontal plane such that the points were not stationary when the distance was measured. Finally, the study is purely an anatomic observational study and research was not conducted regarding the biomechanics, although the aim of study was to provide anatomical theory to aid decision making in the clinic.

## Conclusion

OSLB of the IER has a tough texture and connects with the Stem. The Stem could be used during the Brostrom-Gould procedure, however, we don’t recommend this regardless of the distance between the AFP and the Stem. When the Stem can’t be used for enhancement of a repair operation, other solutions should be explored to protect a newly repaired ATFL.

## Data Availability

The datasets used and/or analyzed during the current study are available from the corresponding author on reasonable request.

## Abbreviations

IER: Inferior extensor retinaculum
Stem: Stem ligament
AFP: Anterior fibular periosteum
OSLB: Oblique Superolateral Band
ATFL: Anterior talofibular ligament
CFL: Calcaneofibular ligament
